# Development and Preliminary Validation of a Questionnaire to Assess Perceptions on the Implementation of UN CRPD Principles Concerning Social Inclusion and Participation of Persons with Disabilities in Municipalities

**DOI:** 10.2174/0117450179360265250113081501

**Published:** 2025-01-16

**Authors:** Samantha Pinna, Massimo Tusconi, Michela Atzeni, Alessandra Perra, Valerio Leonetti, Giulia Cossu, Diego Primavera, Noemi Maria Mereu, Donatella Rita Petretto, Antonio Preti, Elisa Pelosin, Mehmet Eskin, Gian Mario Migliaccio, Mauro Giovanni Carta, Federica Sancassiani

**Affiliations:** 1 Department of Medical Sciences and Public Health, University of Cagliari, 09042 Cagliari, Italy; 2 University Hospital of Cagliari, 09124 Cagliari, Italy; 3 Department of Pedagogy, Philosophy and Psychology, University of Cagliari, 09127 Cagliari, Italy; 4 Department of Neuroscience, University of Turin, Turin, Italy; 5 Department of Neuroscience, Rehabilitation, Ophthalmology, Genetics, Maternal and Child Health, IRCSS Policlinico San Martino, 16132, Genoa, Italy; 6 Department of Psychology, College of Social Sciences and Humanities, Koç University, Rumelifeneri Yolu, 34450 Sariyer, Istanbul, Turkey; 7 Department of Human Sciences and Promotion of the Quality of Life, San Raffaele Rome Open University, 00166 Rome, Italy; 8 Maxima Performa, Athlete Physiology, Psychology and Nutrition Unit, 20126 Milan, Italy

**Keywords:** Social inclusion, Participation, Disability, Validation, Questionnaire, Tool, CRPD, Human rights

## Abstract

**Background:**

The present study aims to evaluate the preliminary content and face validity of the **“**Perceptions on the Implementation of the CRPD in muniCIpalities Questionnaire (PICI-Q),” a self-report questionnaire built to assess the perceptions on how effectively municipalities implement the CRPD principles concerning social inclusion and participation of persons with disabilities in municipalities.

**Methods:**

A two-step Delphi methodology was used to build the questionnaire and assess its content and face validity. A group including health professionals, academics, experts in psychometrics, persons with disabilities and local policymakers was involved in building the questionnaire according to the CRPD articles regarding social inclusion and active participation of persons with disabilities. Two pools of experts assessed the content and face validity and lay stakeholders, respectively.

**Results:**

An average content validity index of 0.95 was obtained, with no items removed. Regarding face validity, all items achieved high scores, ranging from 17 to 21, with a face validity index of 0.95.

**Conclusion:**

The PICI-Q is a promising tool for assessing perceptions of CRPD implementation in municipalities. Its robust preliminary validation suggests it could support local authorities in designing and improving policies and interventions aligned with the CRPD principles of social inclusion and participation for persons with disabilities.

## 1 INTRODUCTION

Although the Convention on the Rights of Persons with Disabilities (CRPD) of the United Nations was adopted in 2006 to “*promote, protect and ensure the full and equal enjoyment of all human rights and fundamental freedoms by all persons with disabilities, and to promote respect for their inherent dignity”* [1] and ratified by 185 countries worldwide, persons with disabilities still face considerable discriminations and inequalities [2–7].

The achievement of several Sustainable Development Goals (SDGs) for persons with disabilities has, in fact, been particularly challenging so far, both in low-and-middle-income countries and in high-income countries, even if policies in line with the principles of the CRPD have been adopted by central governments [8-16]. Compared to persons without disabilities, persons with disabilities are actually more exposed to poverty and hunger (SDGs 1 and 2), natural disasters (SDG 13) and violence (SDG 16) and to the risk of being excluded by the enjoyment of fundamental rights such as that to health, education and work (SDGs 3, 4 and 8), and are less likely to have access to new technologies (ICT), public transport and public spaces and facilities (SDGs 9 and 11). On average, women with disabilities face even more inequalities compared to men with disabilities (SDG 5), particularly in terms of poverty and hunger, access to education, healthcare, employment, opportunities for leadership at all levels of decision-making, and ICT. They are more exposed to the risk of physical and sexual violence and child marriage [2, 8-15].

Considering that 1.3 billion people worldwide (16% of the world's population) experience a significant disability and that their number is going to increase because of a global rise in non-communicable diseases and ageing [17], the implementation of the Convention on the Rights of Persons with Disabilities is a matter of utmost urgency [18-20].

So far, different strategies have been adopted by the other parties that signed and ratified the Convention to comply with the provisions of the CRPD, such as the Strategy for the Rights of Persons with Disabilities 2021-2030 of the European Commission, which highlights the need for a coordinated action at both the national and the EU levels, with a strong commitment from Member States and regional and local authorities [21].

The EU Strategy is only one example of the recognition of the role of local authorities in the implementation of international treaties such as the CRPD. In fact, even if international treaties are ratified by State parties, in recent years, local authorities have increasingly shown their commitment to giving effect to their principles by responding to different global challenges (*e.g*., refugee crises, climate change, *etc*.) and to enforcing international human rights law, also by declaring themselves human rights cities in some cases [22,23]. Both within and outside Europe, a growing number of cities have started to act on the international scene as independent actors and engage with local and international entities, bodies, and processes, for example, by forming or joining city networks (*e.g*., transnational city networks), establishing partnerships with international organizations, or symbolically ratifying international treaties and resolutions, including the Convention on the Rights of Persons with Disabilities [22, 24, 25].

However, even in CRPD-compliant communities, persons with disabilities still face considerable inequalities [26-31], whose persistence highlights the need for local authorities to assess their performance, not only by taking into consideration objective indicators [32] but also the perceptions of the main parties involved in the implementation of the CRPD [33] in their local contexts (municipal policymakers and employees, persons with disabilities, family members of persons with disabilities and representatives of organizations of persons with disabilities) [34-38], to improve their policies, interventions, and services as needed [39-49].

As far as we know, to date, no validated instruments specifically evaluate the perceptions of municipal policymakers and employees, persons with disabilities, family members of persons with disabilities, and representatives of organizations of persons with disabilities on the compliance of their communities with the provisions of the CRPD.

The present paper aims to fill this gap by evaluating the preliminary validity of a self-report questionnaire that assesses the perceptions of municipal policymakers and employees, persons with disabilities, family members of persons with disabilities, and representatives of organizations of persons with disabilities on how effectively municipalities implement the CRPD principles.

## 2 MATERIALS AND METHODS

This study adopts a preliminary validation design to assess the content and the face validity of the ‘Perceptions on the Implementation of the CRPD in municipalities Questionnaire (PICI-Q).’ A two-step Delphi Methodology was employed to develop and validate the questionnaire. The Delphi methodology is a structured process to achieve consensus among a panel of experts on a specific research topic through iterative questionnaires. Experts provide anonymous feedback, and refine their views based on group responses, and the process continues until consensus or stability is reached. This methodology is widely utilized in fields requiring expert judgment to build and validate novel instruments [50–52].

### 2.1 Study Setting

The study was conducted in Sardinia, Italy, as part of the project “Municipalities and Social Inclusion of Persons with Disabilities” funded by the Fondazione di Sardegna and awarded by the Department of Medical Sciences and Public Health of the University of Cagliari, Italy - Unique Project Code (UPC): F73C24000690007. The project aimed to map the actions implemented by local authorities to promote social inclusion and active participation of persons with disabilities in different Sardinian municipalities.

### 2.2 Description of the Questionnaire to Evaluate

The **“**Perceptions on the Implementation of the CRPD in muniCIpalities Questionnaire (PICI-Q)” measures perceptions of how effectively the principles of the United Nations “Convention on the Rights of Persons with Disabilities (CRPD)” are implemented within a municipality (Appendixes **A**, **B**).

This instrument comprises 30 items that correspond to one or more articles of the CRPD, particularly the articles 3, 5, 6, 7, 8, 9, 12, 16, 19, 22, 23, 24, 25, 26, 27, 28, 29, 30, and 33 (Table **1**).

Answers are provided on a five-point Likert scale (“Completely disagree,” “Disagree,” “Neutral,” “Agree,” “Completely agree”) and rated following this scheme: “Completely disagree” scored as 1 point, “Disagree” as 2 points, “Neutral” as 3 points, “Agree” as 4 points, and “Completely agree” as 5 points. All items are positively worded without requiring reverse scoring. Scores on the questionnaire are calculated by summing each participant's responses to the different items. Since the items are ordinal and have at least five categories, they can be treated as an ordinal approximation of a continuous variable [53, 54]. High scores on the questionnaire reflect favorable perceptions regarding the implementation of CRPD principles within a municipality.

### 2.3 Questionnaire Development

The final version of the questionnaire was raised from a two-step Delphi methodology [50, 51] to gain progressive consensus from panels of stakeholders and experts in the field of social inclusion and participation of persons with disabilities. This methodology is indicated to ensure a questionnaire's content and face validity [51, 52].

An instrument is deemed to possess content validity when its development is based on a comprehensive examination of existing data and literature, and an independent panel of subject-matter experts (usually consisting of seven or more members) confirms that the items included are pertinent and accurately reflect the domain under consideration [13, 14]. Face validity is established in a questionnaire when members of the target population concur that it seems to assess the dimension(s) under investigation [55, 56].

Starting from this methodological perspective, the research team reviewed the literature, searching for pre-existing instruments that assessed perceptions of how effectively municipalities implement the principles of the CRPD to promote the social inclusion and participation of persons with disabilities. Subsequently, it selected the articles of the CRPD that pertain to the principles whose implementation may positively impact the social inclusion and active participation of persons with disabilities at the municipal level. Based on these articles, the items were formulated, and a preliminary version of the questionnaire was drafted. Afterwards, a group including health professionals, academics, experts in psychometrics, persons with disabilities, and local administrators was involved in providing general comments on the draft, and all their revisions were incorporated.

### 2.4 Content and Face Validity of the Questionnaire

Content validity was assessed by seven experts in social inclusion and participation of persons with disabilities. This panel examined the questionnaire to ensure it was consistent with its underlying conceptual framework of the CRPD. Experts also evaluated the performance of the items on four dimensions (“item consistency with the content area,” “item wording clarity,” “item perceived easiness,” “item inclusion in the questionnaire”) using a dichotomous response scale (“yes,” scored 1 and “no,” scored 0) [57, 58]. The maximum overall score for content validity was 840 (each of the 30 items of the PICI-Q was evaluated on four dimensions by seven experts, and each dimension could have a maximum score of 1: 30x4x7=840). Based on this score, the average content validity index was calculated for the PICI-Q (dividing the actual overall score for content validity by the maximum overall score for content validity) [59]. The recommended content validity index cut-off value of 0.75 was considered acceptable [59]. Experts had the possibility to provide comments for each item.

Face validity was assessed by seven lay stakeholders (including persons with disabilities and local administrators). The lay stakeholders examined the instruments using a dichotomous response scale (“yes” scored 1 and “no,” scored 0) to evaluate if the items were clear, easy to understand, and relevant [57, 58]. The maximum overall score for face validity was 630 for the PICI-Q (each of the 30 items was evaluated on three dimensions by seven lay stakeholders, and each dimension could have a maximum score of 1: 30x3x7=630). Stakeholders could provide comments for each item.

## 3 RESULTS

### 3.1 Content Validity

Table **2** presents the content validity assessment conducted by the seven experts in social inclusion and participation of persons with disabilities. The first column reflects the evaluation of the items’ consistency, and the second one addresses the clarity of the items, the third one assesses the difficulty of the items, and the fourth one indicates whether the item was considered appropriate for inclusion in the questionnaire. When all seven experts, for example, agreed that an item was consistent, the item received a score of 7 out of 7. The maximum score for each item was 28. When evaluated for the performance of its items, the score range for each item was 0.86-1 (24/28-28/28), with no items excluded, and the questionnaire obtained an overall score of 801/840. An average content validity index of 0.95 was calculated from this score, indicating that the PICI-Q items were overall consistent, clear, and easy to fill.

### 3.2 Face Validity

Table **3** shows the face validity assessment performed by the seven lay stakeholders. The first column reports the evaluation of the items’ clarity, the second one describes if the items were easy to understand, and the third one reports if the item was deemed relevant. The maximum score for each item was 21. The total score of the questionnaire was 601 out of 630, with all items achieving high scores (ranging from 17 to 21). This suggests that the questionnaire was clear, easy to comprehend, and pertinent to the target population.

## 4 DISCUSSION

This study presents the preliminary content and face validation of a recently designed questionnaire, the “Perceptions on the Implementation of the CRPD in muniCIpalities Questionnaire (PICI-Q),” to assess how effectively the United Nations “Convention on the Rights of Persons with Disabilities” (CRPD) principles are implemented within a municipality.

The questionnaire was built by involving a group of stakeholders (*i.e*., health professionals, academics, experts in psychometrics, persons with disabilities, and local administrators) that provided comments and revisions that were addressed by the research team before sending the content and face validation requests to other two groups of experts and lay stakeholders respectively.

The experts affirmed that the questionnaires exhibited strong content validity, with items aligning well with the relevant subject matter being clear, easy to understand, and appropriate for inclusion. Furthermore, face validity received high ratings, as lay stakeholders confirmed that the items were clear, easily comprehensible, and suitable for inclusion. This suggests that the questionnaire effectively addresses the relevant domains through clear and pertinent items. The instrument could be useful in supporting local authorities to develop or improve policies and interventions aimed at ensuring the implementation of the CRPD principles of social inclusion and participation of persons with disabilities within a municipality.

In the case of the CRPD, local authorities' commitment to implementing its principles is, in fact, particularly crucial. Being the closest institutions to citizens and having a clearer picture of the peculiarities of their contexts compared to central governments, they can determine an effective and tangible impact on the lives of persons with disabilities in their communities. However, different factors, including, among others, poor funding, lack of clear guidelines and information from central governments, excessive bureaucracy, limited involvement of organizations of persons with disabilities, inadequate capacity to design effective measures and targeted services, and insufficiency of effective awareness-raising and capacity building activities, often undermine the potential of local authorities to give full effect to the principles of the CRPD and to create inclusive, equal and barrier-free communities [7, 60].

Shaping inclusive communities is undoubtedly a challenging and long process, in which persons with disabilities should be recognized as right-holders [61, 62] and disability as an evolving concept resulting “*from the interaction between persons with impairments and attitudinal and environmental barriers that hinders their full and effective participation in society on an equal basis with others*” [1, 63]. Considering that the concept of “disability” is evolving, thus not fixed, and can vary from society to society depending on the environment, the strategy to implement the principles of the CRPD needs to be context-specific to be effective [64]. In this scenario, local authorities can unquestionably play a key role: thanks to their deep knowledge of the environmental, social, and cultural characteristics of their territories, they can adopt CRPD-compliant measures that respond to the real needs of their communities, and that can be embraced both by persons with disabilities and persons without disabilities [65-67]. In fact, being social inclusion a two-way process, local authorities need to ensure that all citizens play their part and that the attitudinal barriers that lead to stigma and discrimination [68-74] are effectively addressed through measures such as awareness-raising and capacity building [75-80].

Similar efforts have been undertaken internationally, as illustrated by two significant instruments that align with this study’s objectives.

The ITINERIS scale on the rights of persons with intellectual disabilities was developed to assess the extent to which individuals with intellectual disabilities exercise their rights [81]. In alignment with our methodology, a rigorous Delphi methodology involving stakeholders across continents was employed to ensure its relevance and validity [81].

Equally noteworthy is the evaluation of World Health Organization’s QualityRights instruments by Moro *et al*. that assess knowledge, attitudes, and practices related to human rights and mental health, and whose validation demonstrated high reliability and construct validity, offering robust tools for international application to monitor CRPD principles [75].

By comparison, the PICI-Q expands on these frameworks by addressing the municipal context, a critical but underexplored area for CRPD implementation. While the ITINERIS scale and the WHO QualityRights instruments focus on individuals and specific rights holders, the PICI-Q provides a unique lens for assessing institutional commitments and policies within municipalities. This broader scope underscores the pivotal role of local governments in fostering inclusive communities and implementing CRPD principles effectively.

Our study has several strengths. First, it is the first study to rigorously develop and investigate the preliminary validity of a questionnaire to collect perceptions of municipal policymakers and employees, persons with disabilities, family members of persons with disabilities, and representatives of organizations of persons with disabilities on how effectively municipalities implement the CRPD principles. Particularly, content and face validation were conducted using the Delphi methodology to ensure the validity of the questionnaire. Furthermore, persons with disabilities were involved in the questionnaire development and validation phases, coherently with the UN CRPD requirements.

A limitation of this study is that it only addressed the preliminary validation of the PICI-Q in terms of content and face validity. To enhance the robustness of this instrument, future research should examine and verify its construct validity and test-retest reliability.

## CONCLUSION

The **“**Perceptions on the Implementation of the CRPD in muniCIpalities Questionnaire (PICI-Q)” has strong content and face validity, suggesting its potential to assess the implementation of CRPD principles by municipalities effectively. Given the robust preliminary validation, local authorities could promote the use of the instrument to develop or improve policies and interventions aimed at ensuring the implementation of the CRPD principles of social inclusion and participation for persons with disabilities. Further research is needed to verify the construct validity and test-retest reliability of the questionnaire, and broader applicability of the instrument across various municipal contexts.

## Figures and Tables

**Appendix 1:** Perceptions on the implementation of the CRPD in muniCIpalities questionnaire (PICI-Q)” – Italian version.

-	-	Fortemente in Disaccordo	In Disaccordo	Neutrale	D’accordo	Fortemente d’accordo
1.	Nella mia città, le persone con disabilità sono esposte al rischio di discriminazione.					
2.	Nella mia città, le persone con disabilità sono esposte al rischio di maltrattamenti.					
3.	Nella mia città, è facile segnalare violazioni dei diritti umani (salute, lavoro, sicurezza, ecc.) perpetuate nei confronti delle persone con disabilità.					
4.	Nella mia città, i minorenni e le donne con disabilità sono più esposti al rischio di discriminazione rispetto agli uomini con disabilità.					
5.	Nella mia città, l’amministrazione comunale sensibilizza i cittadini al rispetto dei diritti delle persone con disabilità attraverso convegni, manifestazioni pubbliche, cartelloni pubblicitari, post nei social media, ecc.					
6.	Nella mia città, l’amministrazione comunale si impegna a garantire pari diritti e opportunità alle persone con disabilità.					
7.	Nella mia città, la maggior parte dei cittadini rispetta i diritti e la dignità delle persone con disabilità.					
8.	Nella mia città, le persone con disabilità possono crearsi una famiglia, decidendo liberamente e responsabilmente il numero dei figli, senza essere discriminate.					
9.	Nella mia città, l’amministrazione comunale tutela i minorenni con disabilità dal rischio di venire abbandonati, privati delle cure e segregati.					
10.	Nella mia città, l’amministrazione comunale tutela i minorenni dal rischio di essere separati dalla propria famiglia solo a causa di una disabilità sia propria che di un genitore o di un altro componente del nucleo familiare.					
11.	Nella mia città, l’amministrazione comunale garantisce alle persone con disabilità l’esercizio della loro capacità giuridica (es.: prendere decisioni, ereditare proprietà, avere accesso al credito finanziario, sposarsi, avere dei figli, acquistare casa, firmare un contratto di lavoro ecc.…).					
12.	Nella mia città, uffici e luoghi pubblici (es.: scuole, strutture sanitarie, uffici postali, banche, cinema, teatri, musei, monumenti, parchi, ecc.) sono facilmente accessibili dalle persone con disabilità.					
13.	Nella mia città, le strade e i marciapiedi sono facilmente percorribili dalle persone con disabilità, sia in estate che in inverno.					
14.	Nella mia città, i mezzi di trasporto pubblico sono facilmente accessibili dalle persone con disabilità.					
15.	Nella mia città, l’amministrazione comunale garantisce alle persone con disabilità di vivere in case adeguate ai propri bisogni, scegliendo dove e con chi vivere.					
16.	Nella mia città, l’amministrazione comunale garantisce servizi a domicilio, residenziali e altre forme di sostegno per consentire alle persone con disabilità di vivere in modo indipendente.					
17.	Nella mia città, i servizi sociali destinati a tutti i cittadini soddisfano i bisogni delle persone con disabilità.					
18.	Nella mia città, le scuole, le classi o le attività didattiche differenziate per le persone con disabilità sono discriminatorie.					
19.	Nella mia città, l’amministrazione comunale supporta il percorso scolastico e accademico delle persone con disabilità attraverso servizi specifici e sussidi economici.					
20.	Nella mia città, le persone con disabilità accedono facilmente a programmi di orientamento e formazione continua e a servizi per l’impiego.					
21.	Nella mia città, le persone con disabilità possono trovare un lavoro adeguato alle proprie competenze.					
22.	Nella mia città, l’amministrazione comunale sostiene le persone con disabilità che desiderano avviare un’impresa o costituire una cooperativa.					
23.	Nella mia città, le persone con disabilità hanno risorse economiche sufficienti per l’acquisto degli alimenti, dell’abbigliamento e di un’abitazione adeguata ai loro bisogni.					
24.	Nella mia città, le persone con disabilità riescono ad accedere facilmente a servizi sanitari, riabilitativi e preventivi di qualità, gratuiti o a costi accessibili.					
25.	Nella mia città, le persone con disabilità dispongono di dispositivi tecnologici (telefoni cellulari, tablet, computer, ecc.) che garantiscono loro l’accesso all’informazione e alla comunicazione e la possibilità di chiedere aiuto in situazioni di emergenza.					
26.	Nella mia città, le persone con disabilità partecipano attivamente alla vita politica e pubblica (es.: aderiscono a partiti politici, a organizzazioni non governative, si candidano alle elezioni comunali, ricoprono incarichi pubblici, ecc.).					
27.	Nella mia città, le persone con disabilità possono costituire e aderire ad associazioni che tutelano i loro diritti a livello locale, regionale, nazionale, internazionale.					
28.	Nella mia città, le persone con disabilità partecipano attivamente alle attività ricreative, culturali, sportive e legate al tempo libero senza impedimenti.					
29.	Nella mia città, l’amministrazione comunale tutela la privacy sulle informazioni personali e sanitarie delle persone con disabilità.					
30.	Nella mia città, l’amministrazione comunale si impegna a coinvolgere le persone con disabilità nel condurre attività di indagine e di monitoraggio per migliorare l’offerta dei servizi e le politiche a loro dedicate.					

**Appendix 2:** Perceptions on the implementation of the CRPD in muniCIpalities questionnaire (PICI-Q)” – English version.

-	-	Strongly Disagree	Disagree	Neutral	Agree	Strongly Agree
1.	In my city, persons with disabilities are at risk of discrimination.					
2.	In my city, persons with disabilities are at risk of abuse.					
3.	In my city, it is easy to report human rights violations (health, work, security, etc.) committed against persons with disabilities.					
4.	In my city, children and women with disabilities are more at risk of discrimination compared to men with disabilities.					
5.	In my city, the local government raises citizens’ awareness about respecting the rights of persons with disabilities through conferences, public events, billboards, social media posts, etc.					
6.	In my city, the local government is committed to ensuring equal rights and opportunities for persons with disabilities.					
7.	In my city, most citizens respect the rights and dignity of persons with disabilities.					
8.	In my city, persons with disabilities can start a family, freely and responsibly deciding the number of children without facing any discrimination.					
9.	In my city, the local government protects children with disabilities from the risk of abandonment, neglect, and segregation.					
10.	In my city, the local government protects children from being separated from their parents on the basis of a disability of either the child or one or both of the parents.					
11.	In my city, the local government ensures that persons with disabilities can exercise their legal capacity (e.g., make decisions, inherit property, access financial credit, get married, have children, buy a house, sign an employment contract, etc.).					
12.	In my city, offices and public places (e.g., schools, healthcare facilities, post offices, banks, cinemas, theatres, museums, monuments, parks, etc.) are easily accessible for persons with disabilities.					
13.	In my city, persons with disabilities can easily navigate streets and sidewalks, both in summer and winter.					
14.	In my city, persons with disabilities can easily access public transport.					
15.	In my city, the local government ensures that persons with disabilities can live in houses adequate to their needs and choose where and with whom to live.					
16.	In my city, the local government offers in-home and residential support services, and other forms of support to enable persons with disabilities to live independently.					
17.	In my city, social services designed for all citizens meet the needs of persons with disabilities.					
18.	In my city, separate schools, classes, or educational activities for persons with disabilities are discriminatory.					
19.	In my city, the local government supports the education of persons with disabilities, offering specific services and financial aid.					
20.	In my city, persons with disabilities can easily access career guidance programs, continuing training and employment services.					
21.	In my city, persons with disabilities can find jobs suited to their skills.					
22.	In my city, the local government supports persons with disabilities who wish to start a business or develop cooperatives.					
23.	In my city, persons with disabilities have sufficient funds to buy food, clothing, and a house adequate to their needs.					
24.	In my city, persons with disabilities can easily access quality and free or affordable healthcare, rehabilitation, and prevention services.					
25.	In my city, persons with disabilities have access to technological devices (mobile phones, tablets, computers, etc.) that allow them to access information and communication and seek help in case of emergency.					
26.	In my city, persons with disabilities participate actively in political and public life (e.g., join political parties and non-governmental organizations, run in local elections, hold public office, etc.).					
27.	In my city, persons with disabilities can form and join organizations that protect their rights at local, regional, national, and international levels.					
28.	In my city, persons with disabilities participate actively and freely in recreational, cultural, sports, and leisure activities.					
29.	In my city, the local government protects the privacy of personal and health information of persons with disabilities.					
30.	In my city, the local government is committed to integrating persons with disabilities in the performance of surveys and monitoring activities to improve the services and policies that directly affect them.					

**Table 1:** Articles of the CRPD *versus* PICI-Q item(s).

**CRPD Article(s)**	**Item(s)**
Article 3 - General principles	6, 7
Article 5 - Equality and non-discrimination	1
Article 6 - Women with disabilities; Article 7 - Children with disabilities	4
Article 8 - Awareness-raising	5
Article 9 – Accessibility	12, 13, 14, 25
Article 12 - Equal recognition before the law	11
Article 16 - Freedom from exploitation, violence and abuse	2, 3
Article 19 - Living independently and being included in the community	15, 16, 17
Article 22 - Respect for privacy	29
Article 23 - Respect for home and the family	8, 9, 10
Article 24 - Education	18, 19
Article 25 – Health; Article 26 - Habilitation and rehabilitation	24
Article 27 - Work and employment	20, 21, 22
Article 28 - Adequate standard of living and social protection	23
Article 29 - Participation in political and public life	26, 27, 28
Article 33 - National implementation and monitoring	30

**Table 2:** Content validity assessment.

-	**Consistency**	**Clarity**	**Difficulty**	**Inclusion**	**Total Score**
Item 1	7/7	7/7	7/7	7/7	28/28
Item 2	7/7	7/7	7/7	7/7	28/28
Item 3	7/7	5/7	7/7	7/7	26/28
Item 4	7/7	6/7	6/7	6/7	25/28
Item 5	7/7	6/7	7/7	7/7	27/28
Item 6	7/7	6/7	6/7	7/7	26/28
Item 7	7/7	7/7	7/7	6/7	27/28
Item 8	7/7	6/7	5/7	7/7	25/28
Item 9	5/7	7/7	5/7	7/7	24/28
Item 10	7/7	5/7	5/7	7/7	24/28
Item 11	6/7	7/7	5/7	7/7	25/28
Item 12	7/7	7/7	7/7	7/7	28/28
Item 13	7/7	7/7	7/7	7/7	28/28
Item 14	7/7	7/7	7/7	7/7	28/28
Item 15	7/7	7/7	7/7	6/7	27/28
Item 16	7/7	7/7	6/7	7/7	27/28
Item 17	7/7	6/7	6/7	7/7	26/28
Item 18	7/7	6/7	6/7	6/7	25/28
Item 19	7/7	6/7	6/7	7/7	26/28
Item 20	7/7	7/7	7/7	7/7	28/28
Item 21	7/7	7/7	7/7	7/7	28/28
Item 22	7/7	7/7	7/7	7/7	28/28
Item 23	7/7	7/7	6/7	7/7	27/28
Item 24	7/7	7/7	7/7	7/7	28/28
Item 25	7/7	7/7	6/7	7/7	27/28
Item 26	7/7	7/7	7/7	6/7	27/28
Item 27	7/7	7/7	7/7	7/7	28/28
Item 28	7/7	7/7	7/7	6/7	27/28
Item 29	7/7	7/7	7/7	7/7	28/28
Item 30	7/7	6/7	6/7	6/7	25/28
-	207/210	198/210	193/210	203/210	801/840
Content Validity Index	-	-	-	-	0.953

**Table 3:** Face validity assessment.

-	**Clarity**	**Easiness to Understand**	**Relevance**	**Total Score**
Item 1	7/7	6/7	6/7	19/21
Item 2	6/7	6/7	5/7	17/21
Item 3	7/7	6/7	6/7	19/21
Item 4	6/7	5/7	7/7	18/21
Item 5	7/7	7/7	7/7	21/21
Item 6	7/7	7/7	7/7	21/21
Item 7	7/7	7/7	6/7	20/21
Item 8	5/7	5/7	7/7	17/21
Item 9	7/7	7/7	7/7	21/21
Item 10	7/7	6/7	7/7	20/21
Item 11	7/7	6/7	7/7	20/21
Item 12	7/7	7/7	7/7	21/21
Item 13	7/7	7/7	7/7	21/21
Item 14	7/7	7/7	7/7	21/21
Item 15	7/7	7/7	7/7	21/21
Item 16	7/7	7/7	7/7	21/21
Item 17	7/7	6/7	6/7	19/21
Item 18	7/7	6/7	7/7	20/21
Item 19	7/7	6/7	7/7	20/21
Item 20	7/7	6/7	7/7	20/21
Item 21	7/7	7/7	7/7	21/21
Item 22	7/7	6/7	7/7	20/21
Item 23	7/7	7/7	7/7	21/21
Item 24	7/7	6/7	7/7	20/21
Item 25	7/7	6/7	7/7	20/21
Item 26	7/7	7/7	7/7	21/21
Item 27	7/7	6/7	7/7	20/21
Item 28	7/7	7/7	7/7	21/21
Item 29	7/7	6/7	7/7	20/21
Item 30	7/7	6/7	7/7	20/21
-	206/210	191/210	204/210	601/630
Face validity index	-	-	-	0.953

## Data Availability

The data sets used and/or analysed during this study are available from the corresponding author [D.P] upon request.

## LIST OF ABBREVIATIONS

CRPD: = Convention on the Rights of Persons with Disabilities
SDGs: = Sustainable Development Goals
